# Health technology assessment in Brazil: What do healthcare system players think about it?

**DOI:** 10.1590/S1516-31802011000400002

**Published:** 2011-05-05

**Authors:** Marcos Bosi Ferraz, Patricia Coelho de Soárez, Paola Zucchi

**Affiliations:** I MD, PhD. Adjunct Professor, Department of Medicine, Universidade Federal de São Paulo — Escola Paulista de Medicina (Unifesp-EPM), São Paulo, Brazil.; II DDS, MPH, PhD. Professor, Department of Preventive Medicine, Faculdade de Medicina da Universidade de São Paulo (FMUSP), São Paulo, Brazil.; III MD, PhD. Affiliated Professor, Universidade Federal de São Paulo — Escola Paulista de Medicina, São Paulo, Brazil.

**Keywords:** Technology assessment, biomedical, Health facility administrators, Decision making, organizational, Health services administration, Health policy, Avaliação da tecnologia biomédica, Administradores de instituições de saúde, Tomada de decisões gerenciais, Administração de serviços de saúde, Política de saúde

## Abstract

**CONTEXT AND OBJECTIVES:**

The health technology assessment (HTA) process has been developed locally. The aim of this study was to describe, analyze and compare the opinions of participants in international health economics symposia about the HTA process used in Brazil.

**DESIGN AND SETTING:**

Observational cross-sectional study at the 2006 and 2008 International Health Economics Symposia, in São Paulo.

**METHODS:**

A structured questionnaire was applied. For the statistical analysis, the percentage distribution for each category was calculated, and variables were compared using tests for two-sample proportion hypotheses.

**RESULTS:**

Totals of 153 and 74 participants answered the 2006 and 2008 surveys, respectively. The response rate was better for the 2006 survey (67.1%) than for the 2008 survey (31.8%). Most interviewees were between the ages of 30 and 49 years and were managers in the healthcare system. Most of them considered that the current HTA process was incomplete and unable to meet the needs of the healthcare system. They mentioned the government, academia and experts as the three main groups of people who should be involved in the process, and selected efficiency/effectiveness, safety and disease relevance as the three main criteria to be considered in the HTA process. There is a trend towards developing decentralized regionalized HTA processes, with separate assessment and decision-making for the public and private systems.

**CONCLUSIONS:**

The HTA concept is well known. Healthcare system players feel that the process has methodological limitations. Additional surveys are needed to track the HTA process and its application in Brazil.

## RESUMO

**CONTEXTO E OBJETIVOS:**

O processo de avaliação de tecnologias em saúde (ATS) tem sido desenvolvido localmente. O objetivo deste estudo foi descrever, analisar e comparar as opiniões dos participantes dos simpósios internacionais de economia da saúde sobre o processo de ATS utilizado no Brasil.

**TIPO DE ESTUDO E LOCAL:**

Estudo observacional transversal, realizado nos Simpósios Internacionais de Economia da Saúde de 2006 e 2008, em São Paulo.

**MÉTODOS:**

Um questionário estruturado foi aplicado. A análise estatística calculou a distribuição percentual de cada categoria e comparou as variáveis com teste de hipóteses de proporções de duas amostras.

**RESULTADOS:**

Um total de 153 e 74 participantes responderam às pesquisas de 2006 e 2008, respectivamente. A taxa de resposta foi melhor na pesquisa de 2006 (67,1%) do que na pesquisa de 2008 (31,8%). A maioria dos entrevistados estava entre 30 e 49 anos e era composta de gestores do sistema de saúde. A maioria considerou o atual processo de ATS incompleto e incapaz de satisfazer as necessidades do sistema de saúde. Entrevistados mencionaram o governo, academia e especialistas como os três principais grupos de pessoas que deveriam estar envolvidos no processo, e eficiência/efetividade, segurança e relevância da doença como os três principais critérios a serem considerados no processo de ATS. Existe a tendência de desenvolvimento de um processo de ATS descentralizado e regionalizado com avaliações e decisões separadas para o sistema público e privado.

**CONCLUSÕES:**

O conceito ATS é conhecido. Os atores do sistema de saúde acham que o processo tem limitações metodológicas. Pesquisas adicionais são necessárias para acompanhar o processo de ATS e sua aplicação no Brasil.

## INTRODUCTION

The concept of health technology assessment (HTA) made its first international appearance in the 1960s, and has become an important decision-making tool for healthcare managers.^1^ Since the late 1980s, HTA has grown exponentially in Europe, North America, Australia, Latin America and Asia.^2^

In Brazil, HTA started in 1983, with a seminar in Brasilia hosted jointly by the Pan-American Health Organization/World Health Organization (PAHO/WHO) and the Brazilian government. A number of aspects of HTA were discussed at this international event: political issues such as the questionable effectiveness of various healthcare technologies, issues of cost and cost-effectiveness and issues relating to technology transfer.^3^

Over the past six years, the HTA process has developed at an accelerated pace. In 2004, the Ministry of Health created its Department of Science and Technology (Departamento de Ciência e Tecnologia, DECIT) and approved the National Health Policy for Science, Technology and Innovation. In 2005, the General Coordination Office for Health Technology Assessment was created, with the mission of implementing, monitoring and disseminating HTA within Brazil's Unified Healthcare System (Sistema Único de Saúde, SUS). In 2006, procedures for incorporating technology into SUS were created. In 2007, the Brazilian Network for Health Technology Assessment (Rede Brasileira de Avaliação de Tecnologias em Saúde, REBRATS) was created to improve the government's regulatory capacity, and its ability to define prioritization criteria and methodology for HTA studies.^4-6^ In 2009, the Institute for Health Technology Assessment (Instituto de Avaliação de Tecnologia em Saúde, IATS) was created to develop, foster and disseminate HTA in Brazil, thereby providing technical information to help in decision-making processes.^7^ The first national HTA Guidelines for conducting healthcare economic evaluations in Brazil were published in 2009.^8^ In 2011, Brazil is going to host the annual Health Technology Assessment International (HTAi) meeting, in Rio de Janeiro.^9^

Since 2000, the São Paulo Center for Health Economics (Centro Paulista de Economia da Saúde, CPES), at the Universidade Federal de São Paulo (Unifesp), has organized an annual health economics symposium in the state capital. Public and private healthcare professionals, managers and administrators involved in healthcare decision-making participate in the event. Over the years, this symposium has become a major forum of discussion for the main players in the Brazilian healthcare system, i.e. the Ministry of Health, state and municipal departments of health, service providers, healthcare insurers, regulatory agencies, the healthcare pharmaceutical and device industry, patient associations and academics with links to research and teaching institutions.

Every year, the theme for discussion is selected based on its relevance and on the need for a discussion on the topic, among the various healthcare players. The last three symposia (2006, 2007 and 2008) addressed HTA. Domestic and international participants contributed through descriptions of the challenges that they face in HTA processes; the extent to which HTA has helped (or hindered) the decision-making process; and/or the extent to which HTA has in fact improved the healthcare system in their countries.

Although HTA has been incorporated at the federal level, Brazil's regulatory framework is not yet complete. This means that HTA faces a major challenge with regard to its impact on how clinicians and healthcare system decision-makers think and act.^3,10,11^ Regulating healthcare technology has a direct effect on different groups (patients, government, funders, the industry, etc.), and each of these may or may not be favored as a result of the process.^12^ To initiate an awareness-raising process among these individuals, the first step is to understand their thoughts, feelings and expectations regarding HTA.

## OBJECTIVES

In spite of the importance of this topic, little is known about the opinions of Brazilian healthcare players regarding the HTA process. To fill this gap, the aim of this study was to describe, analyze and compare the opinions of participants at the 2006 and 2008 International Health Economics Symposia in relation to the HTA process.

## METHODS

### Type of study, setting and participant sample

This was an observational cross-sectional study that used a structured questionnaire to investigate the opinions of a convenience sample from among the participants at the 2006 and 2008 International Health Economics Symposia.

### Tool

The questionnaire used for this survey was split into two parts:

Part 1 – Participant description. A group of seven questions was used to describe the sample of professionals participating at the International Health Economics Symposium, with regard to the following: gender, age, professional activity within the healthcare system, type of system (public or private) and main segment of professional activity;
Part 2 – Health Technology Assessment. This comprised six questions to ascertain the participants’ opinions on the process used to assess healthcare technology in Brazil: how it is done, who should participate, the criteria to be used and how the media and public opinion influence the process.

### Procedure

The survey questionnaire was applied in a similar way during both the 2006 and the 2008 International Health Economics Symposia, by means of a one-hour interactive session that was included as part of the symposium program. The coordinator of the interactive session presented the purpose of the study and its intended use, and invited the audience to participate. Then, she explained in detail how to use the software (SunVote Professional Voting System, PVS-W52) to answer the questions. A few questions were used as examples to make the participants familiar with the use of the PVS-W52, which is an advanced two-way 2.4G radio frequency technology, with an easy-to-use keypad with 14 keys that is suitable for interactive sessions and audience response. It has multiple functions such as response feedback and number entering, which can be used to collect voters’ polling. The coordinator read out each question, and both the question and the possible answerers were presented on a projection screen simultaneously. The interviewees were asked to give their personal opinion and not the opinion of the sector of activity that they represented. They were asked to press the keypad numbers corresponding to their answer, and had approximately one minute to accomplish this task. A number was assigned to each keypad, so that all the answers would be anonymous. The responses were sent to a collection center that automatically generated an Excel database, and the group results were immediately presented on a projection screen.

### Data analysis

The participants’ responses were analyzed using the Stata 8.0 statistical software. The categorical variables were described in terms of the number of individuals and percentage in each of the categories. Variables were compared using tests for two-sample proportion hypotheses. P values ≤ 0.05 were considered significant.

## RESULTS

Table 1 describes the International Health Economics Symposium participants who responded to the survey in 2006 (n = 153) and 2008 (n = 74). The response rate was better for the 2006 survey (61.7%) than for the 2008 survey (31.8%), with a statistically significant difference between the groups (P = 0.0000). The percentage of women in the sample fell from 55.6% to 41.3% (P = 0.0434). The percentage of participants in the age group between 30 and 49 years fell from 65.3% to 48.6% (P = 0.0181). Nonetheless, most of the participants in both events were between the ages of 30 and 49 years. There were no differences in terms of what type of healthcare system they worked in. In terms of where they performed their professional activities, the number of professionals linked to government or regulatory agencies increased considerably, from 24.0% to 42.6% (P = 0.0055), and the number linked to the healthcare pharmaceutical and device industry fell from 20.8% to 10.3% (P = 0.0011). Most of the respondents primarily held managerial or administrative positions. The number of participants working as providers fell from 13.5% to 8.8% (P = 0.0140), and the number of participants with links to research or teaching institutions increased (P = 0.0140).

**Table 1.** Demographics of the survey participants

	Symposium		
	2006 n (%)	2008 n (%)	P-value
Total number of participants in the symposium	228 (100)	233 (100)	
Total number of participants in the survey	153 (67.1)	74 (31.8)	0.0000
Gender			
Female	84 (55.6)	26 (41.3)	0.0434
Male	67 (44.4)	37 (58.7)	0.0434
Age group (years)			
Under 30	14 (9.3)	10 (14.3)	*
Between 30 and 49	98 (65.3)	34 (48.6)	0.0181
Between 50 and 69	38 (25.3)	25 (35.7)	*
Over 70	-	1 (1.4)	
Healthcare system served by respondent			
Public only	39 (27.7)	24 (36.4)	*
Mostly public	10 (7.1)	5 (7.6)	*
Almost equally divided between public and private	23 (16.3)	10 (15.2)	*
Mostly private	25 (17.7)	7 (10.6)	*
Private only	44 (31.2)	20 (30.3)	*
Location of work			
Government/regulatory agency	35 (24.0)	29 (42.6)	0.0055
University or research and teaching institution	10 (6.9)	10 (14.7)	*
Hospital, laboratory, clinic or support unit	16 (11.0)	4 (5.9)	*
Healthcare operators (self-managed, cooperatives, health insurance, group medicine)	32 (21.9)	13 (19.1)	*
Healthcare pharmaceutical/device industry	45 (30.8)	7 (10.3)	0.0011
Industry or professional association	5 (3.4)	4 (5.9)	*
Other entity not mentioned above	3 (2.0)	1 (1.5)	*
Main role			
Service provider/healthcare	20 (13.5)	6 (8.8)	0.0140
Teaching/research	10 (6.8)	12 (17.6)	0.0140
Administrator/manager	89 (60.1)	39 (57.4)	*
Technician/support	25 (16.9)	9 (13.2)	*
Council/healthcare committee	3 (2.0)	2 (2.9)	*
Student	1 (0.7)	-	
Years working in the healthcare system			
0-5	NA	14 (20.9)	
6-10	NA	14 (20.9)	
Over 11	NA	39 (52.7)	

NA: questions not asked at the 2006 symposium

* Values that did not show a statistically significant difference.

The participants’ opinions about the HTA processes are summarized in Table 2. At both symposia, the majority of the individuals (77.9% in 2006 and 86.8% in 2008) believed that the current HTA processes used in the public and private healthcare systems were methodologically incomplete, insufficient in scope and unable to serve the current needs of the healthcare system. Around 3.1% in 2006 and 7.4% in 2008 stated that the processes were incomplete and broad in scope and unable to serve the current needs of the healthcare system. Only 1.4% in 2006 and 5.9% in 2008 considered that the processes were complete, albeit insufficient in scope and unable to serve the current needs of the healthcare system.

**Table 2.** Participants’ opinions about the Health Technology Assessment (HTA) process in Brazil

Question	Symposium		
	2006 n (%)	2008 n (%)	P-value
1. In your opinion, is the HTA process currently used in our healthcare system (public or private):			
Incomplete (methodologically), insufficient in scope (only for some technologies) and does not meet the needs of the healthcare system?	113 (77.9)	59 (86.8)	*
Incomplete and insufficient in scope, but meets the current needs of the healthcare system?	5 (3.4)	-	
Incomplete and broad in scope; does not meet the current needs of the healthcare system?	19 (3.1)	5 (7.4)	*
Incomplete and broad in scope; meets the current needs of the healthcare system?	2 (1.4)	-	
Complete but insufficient in scope; does not meet the current needs of the healthcare system?	2 (1.4)	4 (5.9)	*
Complete and insufficient in scope, but meets the current needs of the healthcare system?	-	-	
Complete and broad in scope, but does not meet the current needs of the healthcare system?	2 (1.4)	-	
Complete and broad in scope; meets the current needs of the healthcare system?	2 (1.4)	-	
2. Considering that any player in the healthcare system (including all citizens) can suggest which technologies should be assessed, who should participate in the process for selecting which technologies to assess? Mention three or fewer.			
Lay public/society at large	17 (10.8)	15 (23.4)	0.0157
Patients/patient organizations	46 (29.3)	24 (37.5)	*
Researchers/academics	96 (61.1)	41 (64.1)	*
Government and agencies	107 (68.2)	49 (76.6)	*
Professional associations/service providers	61 (38.9)	30 (46.9)	*
Healthcare plan operators	40 (25.5)	11 (17.2)	*
Supplies and products industry	28 (17.8)	6 (9.4)	*
Consumer protection bodies	14 (8.9)	7 (10.9)	*
3. What are the three most important criteria that should be applied to the HTA process?			
Evidence of safety and risk relating to the technology	76 (52.4)	42 (63.6)	*
Evidence of the effectiveness of the technology	117 (80.7)	56 (84.8)	*
High cost of the technology and/or impact on the budget	67 (46.2)	22 (33.3)	*
Relevance of the health problem/impact of the disease (high prevalence, high morbidity, high mortality)	124 (85.5)	51 (77.3)	*
Lack of alternatives for the disease or condition	33 (22.8)	22 (33.3)	*
Legal pressure and/or pressure from society	6 (4.1)	2 (3.0)	*
Technology has never been assessed, even in other countries	6 (4.1)	-	
4. Taking both the public and the private healthcare systems into consideration, should HTA processes:			
Provide a single assessment for both systems followed by a unified decision to incorporate made for both systems?	45 (32.1)	17 (26.6)	*
Provide a single assessment for both systems followed by a decision to incorporate made by each system separately?	55 (39.3)	21 (32.8)	*
Provide separate assessments for each system followed by a unified decision to incorporate made for both systems?	9 (6.4)	5 (7.8)	*
Provide separate assessments and decisions to incorporate made for each system separately?	31 (22.1)	21 (32.8)	*
5. Taking the regional differences in Brazil (population, needs and priorities) into consideration, should the (a) assessment process and (b) decision to incorporate technologies be:			
(a) Centralized and (b) unified, and national in scope?	52 (37.1)	19 (28.4)	*
(a) Centralized with (b) regional decisions?	63 (45.0)	29 (43.3)	*
(a) Regionalized with (b) regional decisions?	25 (17.9)	19 (28.4)	*
6. In your opinion, in general do the media and public opinion favor incorporating new technology in a manner that is:			
Hasty and adequate	5 (3.4)	2 (2.9)	*
Hasty and inadequate	106 (71.6)	48 (70.6)	*
Timely and adequate	14 (9.5)	7 (10.3)	*
Timely and inadequate	5 (3.4)	2 (2.9)	*
Late and adequate	5 (3.4)	3 (4.4)	*
Late and inadequate	13 (8.8)	6 (8.8)	*

* Values that did not show a statistically significant difference.

With regard to who should be involved in the process of selecting the technologies that would be assessed, the responses were similar at the two symposia and mentioned the government and agencies, academic researchers and professional associations as the three main groups that should be involved, followed by the patients. In 2006, 10.8% of the respondents would include the lay public in the process used to prioritize technologies to be assessed, while in 2008, this number jumped to 23.4% (P = 0.0157). As expected, the groups consisting of consumer defense bodies and the healthcare pharmaceutical/device industry received the lowest scores (8.9%-10.9% and 17.8%-9.4%, respectively).

The interviewees selected evidence of effectiveness, relevance of the healthcare problem or impact on disease and evidence of risk and safety associated with use of the technology as the three main factors that should be used to prioritize which healthcare technologies to assess, followed by those relating to high cost of the technology and its impact on the budget (33.3%-46.2%) and lack of alternative for the disease or condition (22.8%-33.3%). Legal pressure and/or pressure from society received the lowest scores (3.0%-4.1%). The answers during the two symposia were similar.

With regard to the assessment process and decisions on whether to incorporate technologies into the public and private healthcare systems, opinions were divided during both symposia. The largest proportion of the 2006 participants (39.3%) felt that the assessment should be the same for both systems and that the decision to incorporate should be made by each system separately. Joint assessment and decisions to incorporate was the option selected by 32.1% of the participants, while 22.1% felt that it would be best if assessment and decisions to incorporate were handled separately by the public and private healthcare systems. In 2008, a large proportion of the participants (32.8%) also selected shared assessment and separate decisions to incorporate, but the same proportion of 32.8% selected separate assessment and incorporation decisions, while 26.6% felt that a single assessment and decision to incorporate should be used for both systems. These findings may suggest that the centralized single assessment process followed by a unified decision for the two systems is moving towards separate assessments and decisions to incorporate for the two systems.

Taking the regional differences in Brazil into consideration, most of the participants (45.0% and 43.3%, respectively) at both symposia suggested that the assessment process should be centralized but that the decision to incorporate healthcare technologies should be regional. In 2006, 37.1% of them chose a centralized unified assessment process and a national decision regarding incorporation, followed by 17.9% that preferred regional processes for both assessment and decisions to incorporate. In 2008, the scenario changed with a decrease in the percentage of those that preferred a centralized unified assessment process and an increase in the percentage of those that preferred regional processes for both assessment and incorporation decisions.

At both symposia, the vast majority of the decision-makers (71.6% in 2006 and 70.6% in 2008) felt that the media and public opinion currently favor incorporating new technologies in a manner that is both hasty and inadequate. Around 10% of them felt that the media and public opinion currently favor incorporating new technologies in a manner that is timely and adequate, and around 9% of them felt that the media and public opinion favored technological incorporation in a late and inadequate manner.

## DISCUSSION

Health Economics Symposia are an important forum for creating awareness and teaching healthcare system players about the dilemmas and challenges of the HTA process. Above all, the forum has provided healthcare leaders and managers with an environment in which to discuss and reflect on the challenges that they face during the decision-making process. It has also promoted a dialogue among researchers and policymakers about how HTA can be a valuable tool for informing the decision-making process in Brazil.

In this study, we used volunteers who were available to us as our sample. The problem with this type of sample is that there is no evidence that such volunteers are representative of the population. Nevertheless, our main objective here was to describe this particular group in an exploratory way. Interviewing these 227 participants provided valuable insights into their opinions on the HTA process, even though this would not yield data on the proportion of healthcare players in the general population who might share these views. In general, the questionnaire responses on the HTA process were similar in 2006 and 2008. These two surveys may suggest rough estimates for the proportion of the population manifesting these opinions. When similar results are obtained repeatedly with different convenience samples, the likelihood that such results apply to the population is greater than when only a single convenience sample is used. Nevertheless, inferences based on such data must be made cautiously because of the possibility of hidden systematic bias.

Fewer participants provided responses in the 2008 (31.8%) survey than did in 2006 (61.7%). When people decline to participate in a study, they may do so for reasons that could be very important for the study results. This is more likely to happen when the study calls for revelation of deeply personal or embarrassing information, which was not the case here. The only difference that might have affected the response rate was the timing of questionnaire application. In 2006, the survey was applied on the first day of the symposium, just after the main lecture of the day. In 2008, the survey was applied in the afternoon of the last day of the symposium. Many participants come from outside São Paulo and often return home before the end of the event. The lower response rate may indicate that the 2008 sample consisted of self-selected participants, people who were motivated to participate, for some reason. Perhaps they were the ones more interested in the HTA process.

The differences do not invalidate the results, which were quite similar for the two symposia. We are confident about the responses provided and believe that this study captured the respondents’ main opinions and made their position regarding the Brazilian HTA process clear.

Increased participation by individuals with links to the government (P = 0.0055) and with links to research and teaching institutions (P = 0.0140) may reflect increased attention to this topic among such individuals, and consequent willingness to participate in the survey as they came to recognize the importance of the topic. It may also be an indication of the importance that both of these groups placed on the HTA process in Brazil. The majority of the assessments conducted to date have been commissioned and funded by the government, and have been carried out by researchers affiliated to local universities.

Regarding the question that explored the HTA process currently used in Brazil, the majority of the participants in both symposia considered that the process was methodologically incomplete. Similar findings were presented in the paper by Pichon-Riviere et al., in relation to whether the 15 key principles for guiding decisions on structure, methods, processes and use of HTA are relevant and useful in Latin America. Decision-makers in 18 Latin American countries, including Brazil, considered that Principle 5 (*HTA should incorporate appropriate methods for assessing cost and benefits*) and Principle 7 (*A full society perspective should be considered when undertaking HTAs*) were very relevant, but they stated that intermediate to large-magnitude "gaps" existed, i.e. mismatches between the importance of HTAs and their real application. This was taken to mean that there was a high level of perceived non-application of these methodological features in HTAs in Latin America, which also perhaps reflected an acceptable level of development of HTA capacity in the region.^13^

The interviewees’ opinion in the present study that the current HTA process was incomplete, insufficient in scope and unable to meet the needs of the healthcare system may be explained by the fact that the development of HTAs, although rapidly growing, is still embryonic in Brazil. Most of the assessments available through the website of DECIT, the Ministry of Health's body responsible for coordinating HTA activities in Brazil, are systematic reviews^5^ and not methodologically complete HTAs. After summarizing the evidence, other methodological steps are necessary to finalize the assessment and make suitable recommendations. An international study looking at the best way to incorporate effective technologies in 12 OECD (Organization for Economic Cooperation and Development) nations showed that only 14% of the HTAs could be categorized as broad assessments of medical technology.^14^ However, although neither complete nor broad, they seem to have presented decision-makers with the information that they need and to have met the goals of their healthcare systems.^15^ To illustrate with an example, the Ministry of Health's Brazilian Bulletin for Health Technology Assessment showed that one systematic review on a product of uncertain clinical usefulness gave rise to a reduction in the annual budget requirement of around 800 million reais in 2005.^6^

The pace at which new knowledge is acquired and the constant and growing offer of new technology, allied with the speed at which information now flows due to modern information and communication media, makes health technology assessment a true challenge to overcome. The need to develop local assessment processes and respect the characteristics of the healthcare system and resource availability pose an additional challenge. Not only is HTA a relatively new process within the international context, and one for which the methodology is still being developed, but also there is a need for qualified people to conduct and critically assess the process, results and recommendations so that HTA may guide the decisions made on behalf of the healthcare system. There is a shortage of technically qualified personnel to perform HTA studies at the national level, which has led to a perception that the process is not broad enough, given that it leaves out numerous technologies.

Thus, although the HTA process flow has existed since 2006, it is still seen as slow, unable to come to a resolution and unable to meet the needs of the healthcare system. The delays and the time required for assessments have also been the subject of criticism in developed nations such as Canada, the UK and Australia, where there are well-established HTA agencies and the process lasts around 11 to 18 months.^16-18^ In general, HTA evaluators and users recognize that the time that it takes to make a complete scientifically sound HTA is paradoxical to the urgent need for information in order to make a decision.^19^ The so-called technical time is considered to be unrealistic and incompatible with the political decision-making time. However, it is important to stress that the quality and scope of the HTA process can be impacted by the volume of resources and time available.^20^ The impact of HTAs depends on timely application in decision-making and subsequent implementation.^21^

In this study, the responses consistently pointed towards the government (Ministry and Departments of Health), researchers and professional associations as the three main groups that should be involved in the process for selecting and prioritizing technologies for assessment. However, in our 2008 survey, we noted that there was greater mention of including the lay public, thereby reflecting what has already happened in other developed nations. For example, international agencies have included all players in their assessment process. In the UK, the National Center for Health and Clinical Excellence (NICE) allows patient groups to participate in all steps involved in developing a recommendation, from designing the scope of the research to including comments in the reports that are issued.^22^ Public interest in both the process and the prioritization of HTAs within decision-making is clear, and a role for "the public" is widely promoted.^23^ The Brazilian HTA policy will have to respond to the challenges of building public involvement into the bodies involved.

The ranking of the three most important elements for assessing healthcare technology reflected the type of information that decision-makers felt was necessary. Between 80.7 and 84.8% believed that effectiveness was an important factor, 77.3-85.5% believed that the relevance of the health problem was important and 52.4-63.6% felt that safety/risk elements were important. These were the same factors as listed in a survey of managers in charge of healthcare funding decisions in OECD countries. In that survey, 91% of the participants felt that safety was an important element, 86% believed that effectiveness was important and 81% felt that the relevance of the healthcare problem or disease burden was important.^20^ It is interesting to note that, although economic issues have always been present in national debates about healthcare funding and sustainability, the high cost of technology and its potential impact on the budget were not selected as one of the three main HTA criteria.

Given the connection between HTA and the bodies that formulate health policy, the structure of the HTA process is molded by the type of healthcare system within which it takes place. In countries in which healthcare is primarily government funded (e.g. Sweden, Canada, Norway, UK and Spain), government agencies have been created for this purpose. On the other hand, in countries with private healthcare systems (such as the United States), HTA is handled by numerous individuals or groups linked to pension funds, healthcare funders or universities.^15,24^

In Brazil, although the healthcare system is mixed (the entire population of 190 million persons has the right to access SUS, while 41 million have access to the private healthcare system), the formal HTA structure is fundamentally linked to the public healthcare system. However, the split in responses to question 4 may demonstrate that there remain questions regarding whether there should be a single assessment process for the two systems. From 2006 to 2008, in spite of an increase in the number of professionals linked to the government or regulatory agencies that would potentially in charge of creating a public assessment body, and a decrease in the percentage of professionals from the pharmaceutical and device industry, who might advocate different assessment processes for the public and supplemental healthcare systems, with less government interference, there was a slight increase in the number of responses selecting different assessments and decisions to incorporate, for the two systems.

This also points towards the potential for tension between, on the one hand, the pharmaceutical and device industry and the providers of private healthcare who wish to offer the newest and best products and services, and thus prefer an HTA process that is quick, and, on the other hand, the SUS public policymakers who, because of lack of funds, must ensure that technologies are incorporated rationally so as to ensure the sustainability of the public healthcare system over time.

## CONCLUSION

The concept of HTA was well-known among leaders and decision-makers. Public and private healthcare system managers felt that the process had methodological limitations and did not fulfill the healthcare system needs. The leaders clearly revealed the main groups who should be involved and the most important criteria that should be applied to the HTA process. There was a trend towards developing decentralized regional HTAs, with different assessments and decisions to incorporate, for the public and private systems.

In spite of the challenges ahead for HTA, and moments of conflict among the various players, it is a tool that can contribute towards building an effective and efficient healthcare system. This study can help in focusing on factors that will make it possible to track the development of the HTA process and its implementation in Brazil.
